# Mutator/Hypermutable Fetal/Juvenile Metakaryotic Stem Cells and Human Colorectal Carcinogenesis

**DOI:** 10.3389/fonc.2013.00267

**Published:** 2013-10-29

**Authors:** Lohith G. Kini, Pablo Herrero-Jimenez, Tushar Kamath, Jayodita Sanghvi, Efren Gutierrez, David Hensle, John Kogel, Rebecca Kusko, Karl Rexer, Ray Kurzweil, Paulo Refinetti, Stephan Morgenthaler, Vera V. Koledova, Elena V. Gostjeva, William G. Thilly

**Affiliations:** ^1^Laboratory for Metakaryotic Biology, Department of Biological Engineering, Massachusetts Institute of Technology, Cambridge, MA, USA; ^2^SLC, Oakville, ON, Canada; ^3^Department of Bioengineering, Stanford University, Stanford, CA, USA; ^4^Massachusetts General Hospital for Children, Boston, MA, USA; ^5^Private Ind., Chicago, IL, USA; ^6^Boston University Medical Center, Boston, MA, USA; ^7^Rexer Analytics, Winchester, MA, USA; ^8^Kurzweil Technologies, Inc., Wellesley, MA, USA; ^9^Department of Mathematics, École Polytechnique Fédérale de Lausanne (EFPL), Lausanne, Switzerland

**Keywords:** stem cells, metakaryotic, mutator, hypermutable, cancer model

## Abstract

Adult age-specific colorectal cancer incidence rates increase exponentially from maturity, reach a maximum, then decline in extreme old age. Armitage and Doll (1) postulated that the exponential increase resulted from “*n*” mutations occurring throughout adult life in normal “cells at risk” that *initiated* the growth of a preneoplastic colony in which subsequent “*m*” mutations *promoted* one of the preneoplastic “cells at risk” to form a lethal neoplasia. We have reported cytologic evidence that these “cells at risk” are fetal/juvenile organogenic, then preneoplastic *metakaryotic* stem cells. Metakaryotic cells display stem-like behaviors of both symmetric and asymmetric nuclear divisions and peculiarities such as bell shaped nuclei and amitotic nuclear fission that distinguish them from embryonic, eukaryotic stem cells. Analyses of mutant colony sizes and numbers in adult lung epithelia supported the inferences that the metakaryotic organogenic stem cells are constitutively mutator/hypermutable and that their contributions to cancer initiation are limited to the fetal/juvenile period. We have amended the two-stage model of Armitage and Doll and incorporated these several inferences in a computer program CancerFit v.5.0. We compared the expectations of the amended model to adult (15–104 years) age-specific colon cancer rates for European-American males born 1890–99 and observed remarkable concordance. When estimates of normal colonic fetal/juvenile *APC* and *OAT* gene mutation rates (∼2–5 × 10^−5^ per stem cell doubling) and preneoplastic colonic gene loss rates (∼8 × 10^−3^) were applied, the model was in accordance only for the values of *n* = 2 and *m* = 4 or 5.

## Introduction

The age-specific incidence of colorectal cancer is here reconsidered in terms of stem cell growth and oncomutation in normal stem cells of the fetal/juvenile period (tumor initiation) and thereafter in the preneoplastic stem cells of colonic adenomas (tumor promotion) from which lethal colonic adenocarcinomas arise. This reconsideration was prompted by discoveries concerning *metakaryotic* stem cells in organogenesis and carcinogenesis (2–5) and by evidence that normal organogenic stem cells experience unexpectedly high rates of genetic changes required for tumor initiation (6).

### Overview of organogenesis and carcinogenesis

The human body arises from a single fertilized egg and in a series of cell divisions creates a body mass of some 2^44^ cells at maturity. However, these cells are not homogeneous but are apportioned among the organs each containing several distinct tissue layers containing in turn one or more histologically recognizable cell types. The epithelial layers of many solid organs, such as those of the gastrointestinal tract, the lung, breast and prostate are of special interest because it appears that the vast majority of lethal tumors arise from these layers. Each of these epitheloid or adenocarcinomatous (gland-like) tumors displays histological organization that, however distorted, resembles the histological organization of that organ during fetal growth and development (7). In the case of the human colon, histologic alterations at multiple foci in the embryonic mid- and hind gut begin at about the 5th week of gestation; by the 25th week one may observe the parallel array of crypts that open to the colonic lumen numbering about one million (∼2^20^) in a newborn and about ten million (∼2^23.25^) at maturity with each crypt containing about two thousand (∼2^11^) epithelial cells.

In adult epithelial tissues slowly growing epitheloid colonies (adenomas) are observed from which more rapidly growing colonies emerge (adenocarcinomas). From some, but not all, adenocarcinomas even more rapidly growing metastases emerge that are distributed throughout the body. It appears that each succeeding step represents an event in a single stem cell such that each form of lesion is clonal.

### Metakaryotic stem cells of organogenesis and carcinogenesis

Embryonic stem cell lines derived from human blastulas are eukaryotic cells. They have spheroidal nuclei enclosed in a nuclear membrane that exists as an organelle within the cell cytoplasm. Their DNA content doubles in an S-phase that is completed a few hours before the condensation of chromosomes in prophase marks the beginning of mitosis by which the two sister genomes are segregated during cell fission to form two sister cells.

But beginning in the fourth- to fifth-week of gestation and in all preneoplasias and neoplasias examined a different kind of cell is found in which the genomic DNA is contained in a large, hollow, bell shaped structure. These appear to arise from precursor cells with spherical nuclei, i.e., resembling eukaryotic embryonic stem cells, in which a belt of condensed chromatin marks the beginning of an amitotic process in which two separate facing hemispheres are created each containing the diploid amount of DNA. Soon these bell shaped nuclei are enclosed in a sarcomeric pod or tubular syncytium (3, 4). In the ∼5th–12th week of gestation the number of bell shaped nuclei per tubular syncytium increases by a form of symmetric amitosis resembling one paper cup separating from another. The number of syncytia increases rapidly and distribute non-randomly in space and time within fetal meta-organs up to about the 12th week. Then the syncytia disappear but the bell shaped nuclei persist as mononuclear cells with oblate spheroidal mucinous cytoplasms. These continue to increase by symmetric cup-from-cup amitoses without any detectable condensation of chromosomes. Bell shaped nuclei are appended to, rather than enclosed in, the cytoplasmic organelles. DNA doubles not in an S-phase preceding nuclear fission as in eukaryotes but during and after amitotic segregation of bell shaped sister nuclei (2–4). The genomes of bell shaped nuclei between doublings appear to be organized in a set of circular structures each containing one or more homologously paired chromosomal elements specifically end-joined at their telomeres (5).

Both in the syncytial and post syncytial period of development bell shaped nuclei of the developing colon undergo asymmetric amitoses in which any of at least nine distinct morphologic forms of closed nuclei emerge from bell shaped nuclei. These subsequently undergo a series of DNA doublings and mitoses that create the eukaryotic cells that populate the crypts of the colonic epithelium throughout the second and third fetal trimesters and the juvenile period. Cells with bell shaped nuclei external to cytoplasmic organelles are located at the bases of colonic crypts both in the developing fetus and colonic adenocarcinomas. After maturity cells with bell shaped nuclei are rarely found at the base of colonic crypts or epithelia of any organs observed to date but are invariably found in incipient micro-colonies in colonic adenomas and at the base of crypts in colonic adenocarcinomas and their derived metastases (3).

Insofar as these cells displayed cytologic behaviors distinguishing them from eukaryotic cells, they were designated as *metakaryotic* cells because they were first observed in the formation of meta-organs during the 5th- to 12th-week of gestation (3). Their discovery in the development of many organs including those of the developing digestive and nervous systems and derived tumors as well as their appearance in a developing plant marked them as an important and general part of organogenesis and carcinogenesis with evolutionary origins preceding the separation of plants and animals (4).

The demonstration that the bell shaped nuclei of metakaryotic cells underwent both symmetric amitoses to increase in number and asymmetric amitoses to form the mitotic parenchymal cells of the organ or tumor marked them as organogenic or carcinogenic stem cells (2–4). These observations confirmed and extended the observations and interpretations of Child (1907) that the gonadal development of a sheep tapeworm involved a series of amitotic divisions followed by a mitotic expansion and directly contradicted the opinion of Boveri (8) that tissue development and tumor growth depended solely on mitotic fission.

### High rates of stem cell mutation in a developing human organs

Measurement of five specific nuclear point mutations and 17 specific mitochondrial point mutations in the DNA of micro-anatomical samples of adult human lung epithelia revealed unexpectedly high numbers of somatic mutations in the *TP53*, *KRAS*, and *HPRT1* nuclear genes and in the mitochondrial genome (bp10,100–10,101) (6, 9). Plotting mutant colony numbers as a function of colony size for the lung data revealed a Luria–Delbruck distribution (10). This finding indicated that the mutations occurred at a constant rate per organogenic stem cell doubling during the exponential growth of the organ, i.e., the fetal/juvenile period. Furthermore, mutant fractions in the lung epithelium did not increase with age indicating that mutation rates in maintenance stem cells of lung turnover units were much lower than in organogenic stem cells. Consistent with this interpretation was the observation that overall lung mutant colony numbers of any size did not significantly increase with age in adults. Using these data we estimated a gene-inactivation mutation rate per lung stem cell doubling of about 2–4 × 10^−4^ for the *TP53* and *HPRT1* genes.

Histological enumeration of colonic polyps carrying a somatic mutation of the *APC* gene in FAP patients’ colons (11) and of colonic crypts carrying a somatic mutation in the sialomucin acid *O*-acetyl transferase (*OAT)* gene (12) provided data that have allowed us to estimate that gene-inactivation mutation rates for stem cells of the developing colon are about 2–5 × 10^−5^. As the gene-inactivation rate of human B-cells grown in culture has been estimated as ∼2 × 10^−7^ per cell doubling (13) it is clear that fetal/juvenile stem cell mutation rates are some 100 (colon) to 1000×(lung) higher than previously associated with human eukaryotic cells. Levels of loss of heterozygosity for polymorphic markers in colonic preneoplasia of ∼0.25 allow estimation of preneoplastic gene deletion rates of about 8 × 10^−3^ per gene copy per preneoplastic stem cell doubling (14). Based on these observations we inferred that high rates of genetic change or “genomic instability,” generally associated with preneoplastic and neoplastic growths, are a characteristic of the metakaryotic stem cells of human organogenesis.

## Materials and Methods

### Biologically based algebraic cancer models

We have incorporated these new biological findings/inferences in a revised model of carcinogenesis adopting and extending the two-stage model first proposed and expressed in algebraic form by Armitage and Doll (1): “*n*” *initiating* mutations in an organogenic metakaryotic stem cell permit it to (a) avoid metamorphosis to a non-growing maintenance stem cell at maturity and (b) continue to grow at approximately the juvenile growth rate as a preneoplastic colony; “*m*” *promoting* events in a preneoplastic metakaryotic stem cell permit it to form a rapidly growing lethal neoplastic colony.

The model so revised permits, for the first time, the use of key parameters derived from clinical observations: the number of stem cell doublings during organogenesis (*a*_max_) and the rates per metakaryotic organogenic stem cell doubling of *n* events necessary for initiation (*R*_i_, *R*_j_, …, *R*_n_) and the rates per preneoplastic metakaryotic stem cell doubling of *m* events necessary for promotion (*R*_A_, *R*_B_, …, *R*_m_). Comparison of quantitative predictions based on the revised model to the age-specific incidence rate for that cancer in a population in which data are available for the entire adult life span of ∼15–104 years tests the general accuracy of the assumptions incorporated in the model.

#### One-stage models

Cohnheim inferred from similarity of histologic organization between second trimester fetuses and adenocarcinomas that tumors originated in cells responsible for embryonic/fetal growth and differentiation (7). Boveri inferred from the presence of altered chromosome numbers and structures in some primary tumors and metastases that tumors involved chromosomal/genetic changes (8). In the early 1950s several theorists sought to reconcile the increasing rate of cancers with adult age in terms of new understandings of genetics and genetic change particularly the demonstration that human tumors were of clonal origin. They posited that any single cell at risk in a static, adult cell population could be created from normal “cells at risk” by “*n*” required genetic events (15– 17). In particular, Armitage and Doll (17) examined human age-specific mortality rates, OBS(*h,t*) for several cancer sites and noted a relationship, limited to the age range of 25–75, of the form log OBS(*h,t*) = log *K t*^(^*^n^* ^− 1)^ = log *K* + (*n* − 1)log *t*, where “*n*” was the number of required oncogenic events in a constant number of cells at risk.

This treatment, presented as a hypothesis by the authors, suggested that *n* might be 5, 6, or 7, the source of the idea that such numbers of oncomutations are required in human carcinogenesis (17).

#### Two-stage models

However, the “one-stage” models did not account either for growth of stem cell numbers in development or for clinically observed slowly growing preneoplastic lesions such as colonic adenomas. Platt was apparently the first to ask if early mutations could create a cell population with a growth advantage in which later necessary oncomutations could occur (18). Armitage and Doll responded to Platt’s suggestion by testing a model in which a single normal “cell at risk” was *initiated* by “*n*” required events could give rise to an exponentially growing preneoplastic colony in which any “cell at risk” was *promoted* to a tumor forming cell by “*m*” independent events (1). They chose the words *initiation* and *promotion* from the findings in experimental animal studies that certain chemical treatments could create a latent capability of tumor formation (initiation) that subsequent chemical treatments could provoke into growth of a visible neoplasia (promotion) (19). Their reasoning may be represented as log OBS(*t*) = log *Le*^−μ^*^t^* = log *L* + μ*t* where *L* is a constant related to the product of initiation and promotion mutation rates and μ is the annual growth rate of preneoplastic cells at risk in adult tissue. Initiation events were posited to occur at constant annual rates per person from birth through old age. Human populations were considered to be homogeneous with regard to risk. It was noted that initiated cells at risk “had sufficient selective advantage to double in number about every 5 years” (1). They noted that both the “one-stage” and “two-stage” models could be fit by judicious choice of parameter values to age-specific mortality data for the age of death interval 25–75 years and, therefore, they could not exclude either possibility on this basis alone.

#### Limitation of “two-stage” model: declining cancer rates in extreme old age

**Figure 1:**
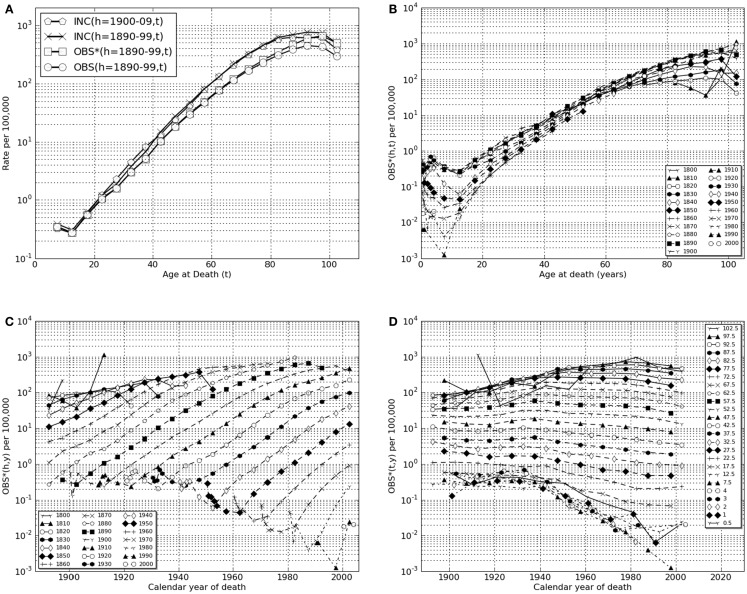
**Lower gastrointestinal cancer in European-American males, calendar years of death, 1890–2006**. (**A)** Transformations linking raw mortality rates, OBS(*h,t*), with estimates of incidence rates, INC(*h,t*). Shown are the raw mortality rate function, OBS(*h,t*), for *h* = 1890–99 corrected for coincident forms of death which becomes the function OBS*(*h,t*) which, in turn, corrected for historically increasing survival rate, becomes the incidence rate function, INC(*h,t*). Also shown is INC(*h* = 1900–09, *t*). **(B)** OBS*(*h*,*t*), coincidence corrected death rates for birth cohort intervals, *h* = 1800–09, …, 2000–06, vs. age intervals of death, “*t*” = 0.5, 1, 2, 3, 4, 5–9, …, 100–104. **(C)** OBS*(*h*,*y*), coincidence corrected death rates for birth-decade intervals, *h* vs. calendar year of death intervals, “*y*” = 1900–1904, …, 2000–2004, 2005–2006. **(D)** OBS*(*t,y*), coincidence corrected death rates for intervals of age at death “*t*” vs. calendar year of death intervals, “*y*.”

However, neither the one-stage nor two-stage cancer models accounted for age-specific rates outside of the interval of 25–75 years. The rates of most forms of cancer decline from the first years of life to a minimum in mid-juvenile years, increase sub-exponentially into old age, reach a maximum then decline in extreme old age (Figure 1) (14, 20). Doll’s group ascribed the apparent maximum raw mortality rate in old age to either under-diagnosis of cancer in old age or the presence of a subpopulation that was not at risk (21). We algebraically amended the basic two-stage model using the suggestion of to account for a subfraction of the population, *F*, could be at risk for all of the events required for carcinogenesis in a particular organ, while the subfraction (1 − *F*) was not at risk. This modification resulted in improved fits of prediction to observed incidence rates for colorectal cancer in the United States in old and extreme old age (14, 20). Predictions even more closely related to cancer incidence data may be expected if explicit partitioning of the general populations into subpopulations differing in quantitative risk factors are introduced, e.g., oncomutation rates (6), preneoplastic growth rates, and even individual size at maturity. Other biological hypotheses exist. For instance, it has been suggested that senescence itself suppresses preneoplastic or neoplastic growth (22, 23).

#### Initiation

##### Kinds and number of initiation mutations, *n*

More than 50 years after Armitage and Doll’s pioneering postulates, the number, nature, and origins of oncomutations that *initiate* the slow growth of preneoplastic lesions and later *promote* a preneoplastic cell to the rapid growth of neoplastic lesions remain obscure for most common cancers, e.g., lung, breast, prostate, pancreas. For a few common cancer types, initiation events have been convincingly associated with the loss of both maternal and paternal alleles of specific autosomal tumor suppressor genes, e.g., colorectal (*APC*), kidney (*VHL*), nervous system (*NF1*, *NF2*), and basal skin (*PTCH*) cancers (24). Accordingly, we chose colorectal or “lower GI tract” cancer as a first example. The inherited early onset syndrome of multiple adenomatous polyps in the lower GI tract (colon and rectum), familial adenomatous polyposis coli or FAP, usually arises from inherited heterozygosity of the *APC* gene. Loss of the second *APC* allele represents a necessary and possibly sufficient event for *initiation* followed by appearance of a growing adenomatous polyp as a preneoplastic lesion, i.e., the number of required colonic initiating mutations is equal to, or greater than, 2.

The deductions of Knudson (25) based on observation and analysis of certain inherited pediatric cancer syndromes such as Wilm’s tumor and retinoblastoma had previously indicated that two mutations in an organogenic lineage were necessary and sufficient to cause these diseases. A two-stage cancer model explicitly positing *n* = 2 was subsequently developed (26). Others motivated or dismayed by the large number and kinds of genetic and epigenetic changes displayed by most, but not all, cancers have postulated that a much greater number of successive events are required (27, 28).

##### Limitation to the period of organ development

Influenced by studies and models of mutation in bacterial populations (10) two cancer modelers independently noted that the age-specific exponential increase in cancer rates in adults might have origins in the approximately exponential increase in organ cell number and, thus, initiated cell number from the beginning of organogenesis to maturity (29–31). A two-stage model of colon cancer incorporating mutation in juvenile (but not fetal) growth periods yielded expectations in concordance with observation (14, 20). A general, untested assumption that fetal mutation rates would/should be very low seems to have blocked further examination of the possibility that fetal mutation was a strong driver of tumor initiation. This idea was dispelled by direct assay at the DNA level of point mutations in micro-anatomical samples of upper bronchial epithelium from 15 healthy human lungs (6, 32).

In this present effort addressing mutations and cancers of the colon we have had recourse to literature reports from which it is possible to obtain reasonable first estimates of fetal/juvenile mutation rates in development of that organ. For instance, patients heterozygous for the *APC* gene are reported to have thousands of adenomatous polyps in adulthood presumably resulting from a single mutation inactivating the single active inherited *APC* allele. The inherited condition appears to be fully penetrant: all known *APC* heterozygotes have displayed multiple adenomas and, if untreated, adenocarcinomas. It should also be noted that the number of adenomatous polyps varies widely within family members afflicted with FAP, an observation extended to mutation rates in human lung epithelium that were found to vary about sevenfold from upper to lower decile estimates (6, 11, 24, 32).

Here we regard polyp number as a probable underestimate of actual mutated stem cells as many patients die before polyps arising late in juvenile development could be recognized. Allowing for 5000–10,000 potential polyps from the stem cells forming about 10^7^ colonic crypts suggests that events per stem cell doubling (∼23.25 to create the crypt number observed) would be of the order of 2–4 × 10^−5^ gene-inactivating events per stem cell doubling for the *APC* gene. These events would include point mutations, chromosomal, and other possibilities including reciprocal recombinations. Our derived estimate of the geometrical mean rate of loss of the second allele of *OAT* in colons of heterozygotes is also ∼2–4 × 10^−5^ (12). We later use these estimates to compare initiation mutation rates predicted for different possible numbers, “*n*” of required initiation mutations.

##### Nature of cells in which initiation mutations occur

Confusion arises in the use of these models in that the earliest efforts treated the number of “cells at risk” of initiation or promotion as constant (15–17) while the 1957 effort of Armitage and Doll treated initiation as occurring in a constant number of cells at risk while promotional mutations occurred in a growing population of initiated cells (1). Normal “cells at risk” might have included only stem cells or any of the stem plus non-stem cells of an epithelial maintenance turnover unit (14, 20). Here we explore the possibility that normal “cells at risk” are solely metakaryotic stem cells (3, 4) that may be readily detected and counted in growing tissues, preneoplastic, and neoplastic lesions. Of course, there are other possibilities, the study of human organogenic and carcinogenic stem cells is still in its own gestation period.

#### Promotion

Clinical observations have shown that preneoplastic lesions of several organs continue to grow after maturity and give rise to lethal neoplasias through extreme old age. Promotional genetic changes or other events, if any, may therefore be hypothesized to occur throughout adult life in preneoplastic colonies in either symmetric (net growth) or asymmetric (turnover) amitoses of initiated metakaryotic stem cells. Uncertainties abound regarding the process called “promotion.”

First, it must be emphasized that no genetic or epigenetic events have yet been found that fulfill the logical requirements for a rare human promotion event. Secondly, as pointed out by Peto, mathematical analyses of non-linear age-specific incidence functions alone cannot define the number of “*n*” initiation or “*m*” promotion mutations using Armitage and Doll’s one-stage or two-stage models (33).

#### Progression

The steps toward deaths from primary neoplasias such as colonic adenocarcinomas and/or their metastases may involve one or more additional mutations/events in a stem cell during primary tumor growth. Here we accept the approximation of ∼2.5 years between the last required promotion event and death (1).

### Using mortality and survival data to estimate age-specific incidence, INC(*h*,*t*)

#### U.S. age-specific cancer mortality rates (1900–2006)

Trial of any model of age-specific cancer experience requires a robust set of data to which predictions may be compared. The MIT-administered website^1^ provides researchers with age-, gender, ethnicity-, and birth cohort-specific population and mortality data organized as Excel^®^ files with several different forms of tabular and graphic summaries. Data are provided as recorded annually from 1900 through 2006 in the USA for most common cancer types and other major forms of mortality such as cardiovascular and cerebrovascular diseases. (Data for Japan are provided for most forms of cancer from 1952 through 1996.) These data permit examination of disease-specific mortality rates for the entire United States throughout the adult lifetimes (15–104 years) of persons of the same birth year or decade cohort born from ∼1885 to 1910. Earlier birth decades lack data that were unrecorded before 1900. Later birth decade cohorts have not yet reached extreme old age.

Many forms of cancer such colorectal, pancreatic, and brain cancers as well as vascular diseases and Type II diabetes display a sub-exponential increase in mortality rates from maturity to a maximum then declining rate in extreme old age. The calculated model presented here, CAL(*h,t*) is aimed at these forms of disease. (However, cancers of other organs including pharynx, tongue, testes, ovaries, uterus, and breast display a clearly different age-specific mortality rate function in which a distinctly more rapid rise in age-specific mortality rates are observed in young than in middle to older aged adults.) Data for many cancer sites are further limited because U.S. national recording began later than 1900, e.g., lung or pancreas in 1930.

This database is updated using ASCII files from the National Center for Health Statistics. U.S. data is organized by gender and major ethnic groups: European Americans and Non-European Americans, the latter comprising primarily African Americans. Information for years 1900–1991 were manually transcribed and re-organized from successive volumes of Vital Statistics of the United States published by the U.S. Census Bureau (1900–1935) and then by the U.S. Public Health Service (1936–1992) (Pablo Herrero-Jimenez), from digital versions of the same publication (1992–1997) (Efren Gutierrez) and directly from comprehensive ASCI files since 1998 (Karl Rexer, Rebecca Kusko, Lohith G. Kini). The computer program Cancer Fit v.5.0 described below interfaces with downloaded Excel^®^ files from this data set.

As originally organized by the U.S. Census Bureau, the number of deaths for each form of mortality were recorded in each calendar year, *y*, for cohorts defined by gender, ethnicity, and age of death intervals, *t* = 0, 1, 2, 3, 4, 5–9, …, 100–104 years. In 1900 the two major ethnic designations were “white” and “non-white.” As “white” included families immigrating from Europe or through former Spanish, French, and Portuguese colonies of North and South America the classification “white” was denominated as European-American [EAM (male), EAF (female)] and the “non-white” consisting primarily of families of African descent was designated Non-European-American [NEAM (male), NEAF (female)].

#### Transforming raw mortality data to incidence estimates

Any single recorded death included the (a) recorded primary cause of death, (b) gender, (c) ethnicity, (d) age at death, “*t*” (e) year of death, “*y*,” and allowed calculation of (*f*) year of birth, “*h*.” Age-specific mortality from a defined disease effecting a specific gender and ethnic group here characterized by the three related numbers, year of birth (*h*), age at death (*t*), and calendar year of death (*y*) such that *t* = *y* − *h*. Biases such as misdiagnosis are recognized but beyond the scope of this treatment.

Thus the data of annual number of deaths for lower GI tract cancer mortality in European-American males from 1900 to 2006 may be represented as MOR (EAM, lower GI tract, *y*, *t*, *h*). Since this paper will use only data for lower GI tract cancer in the EAM group we will shorten this designation to the form MOR(*y*,*t*,*h*).

It is possible to group data entries into defined intervals of calendar birth or death years or age of death as desired. Herein group data from age of birth decades such that the grouped variable, *h*, has values of 1800–1809, 1810–1819, …, 1990–1999, 2000–2006 (see text footnote 1). Similarly the calendar years of death may be grouped such that the variable representing grouped calendar years of death, *y*, has values of 1900–04, 1905–09, …, 1995–1999, 2000–2004, 2005–06. And the grouped variable for age of death, *t*, has values of 1, 2, 3, 4, 5–9, 10–14, …, 95–99, 100–104.

For studies of age-specific cancer rates, the data are organized as birth year cohorts and age of death intervals as, for instance, MOR(*h,t*) in which the grouped mortality data for each birth-decade cohort, e.g., 1890–99 would be plotted as a function of age of death intervals in adult life, *t* = 15–19, …, 100–104.

The raw data from which all other variables are derived are the number of deaths reported each year, MOR(*h,t*), and the reported population size, POP(*h,t*). Their ratios, OBS(*h,t*) serve as a raw estimate of age-specific (*t*) death rates (deaths per person alive within the age interval) for each defined birth cohort (*h*).

However, historically high death rates from other causes among the youngest and oldest members of the population automatically lead to significant underestimation of persons who would have died of the disease observed because they died within the interval of another cause. To overcome this bias we needed a correction factor for each age of death interval of each birth cohort. To provide this we defined the set of values for deaths by all causes (total deaths) in each age of death interval as TOT(*h,t*) = MOR[all causes, (*h,t*)/POP(*h,t*)]. We reasoned that a first order correction for coincident forms of death could be accomplished by defining a coincidence corrected age-specific mortality rate, OBS^∗^(*h,t*):

(1) OBS(h,t)=MOR(h,t)∕POP(h,t)

(2) TOT(h,t)=MOR(all causes)(h,t)∕POP(h,t)

(3) $${\text{OBS}}^{*}\left(h,t\right)=\mathrm{OBS}\left(h,t\right)∕\left[1-\mathrm{TOT}\left(h,t\right)+\mathrm{OBS}\left(h,t\right)\right]$$

In Table 1 we show the exact steps used to define OBS^∗^(*h,t*) for the birth-decade cohort *h* = 1890–99 for the age of death interval *t* = 100–104 years for lower GI tract cancer in European-American males. Here it may be seen that the estimate of OBS^∗^(*h,t*) = 501 × 10^−5^ is derived as shown from 10 separate estimates of the number of recorded deaths, MOR(*h* = 1890, …, 1899, *t* = 102) and corresponding 10 separate population values, POP(*h* = 1890, …, 1899, *t* = 102) corrected for coincident deaths within each year by the values of TOT(*h* = 1890, …,1899, *t* = 102).

**Table 1:** **Arithmetic steps in definition of OBS^∗^(*y* = 1992, …, 2001; *t* = 100–04 years), from reported deaths, MOR(*y,t*) for lower GI tract cancer and population sizes, POP(*y,t*) for EAM**.

Year of death (*y*)	Year of birth (*h*)	MOR(*h* = 1890–99, *t* = 100–104)	POP(*h,t*)	TOT(*h,t*)	OBS(*h,t*) = MOR(*h,t*)/ POP(*h,t*) × 10^5^	OBS*(*h,t*) = OBS(*h*,*t*)/ (1 − TOT(*h,t*) + OBS(*h*,*y*) × 10^5^
1992	1890	27	4656	0.44	580	1035
1993	1891	19	6281	0.35	303	466
1994	1892	19	6484	0.36	293	458
1995	1893	18	6847	0.36	263	411
1996	1894	24	7273	0.34	330	500
1997	1895	20	7642	0.36	262	409
1998	1896	25	7682	0.32	325	478
1999	1897	22	7719	0.32	285	419
2000	1898	23	7753	0.32	238	350
2001	1899	30	8272	0.29	363	511
1992–2001	1890–1899					501

This is the age-specific death rate corrected for coincident deaths in the same reporting years, *y*. Applies eqs 1, 2, and 3. Note that these data comprise the set used to define the value of OBS*(*h* = 1890–99, *t* = 100–04) for the birth-decade cohort of 1890–99 dying from colorectal cancers in the 100–104 age interval, the mid year age being 102 years: 1992–102 ⇒ 1890, 1993–102 ⇒ 1891, …, 2001–102 ⇒ 1899.

#### Accounting for historical improvements in colorectal cancer treatment

Given the values of OBS^∗^(*h,t*) one must account for the fact that a correctly diagnosed tumor of the lower GI tract would not necessarily be mortal. There is convincing evidence that the probability of surviving at least 5 years after competent diagnosis, SUR(*h,t*), has been rising over the last ∼70 years (14, 20). The values of OBS^∗^(*h,t*) derived from deaths alone must represent an underestimate of the age-specific disease incidence, defined here as the fraction of persons at a given age in which the disease first appears, INC(*h,t*). If the values of SUR(*h,t*) are available, the estimate of disease incidence may be improved by calculating INC(*h,t*) = OBS^∗^(*h,t*)/[1 − SUR(*h,t*)].This step is illustrated in Table 2 in which the values of OBS^∗^(*h,t*) are transformed into values of INC(*h,t*) by use of historical 5-year post-diagnosis survival estimates for lower GI tract cancer (14).

(4) $$\mathrm{INC}\left(h,t\right)={\text{OBS}}^{*}\left(h,t\right)∕\left[1-\mathrm{SUR}\left(h,t\right)\right]$$

**Table 2:** **Arithmetic steps in definition of INC(*h,t*), the age-specific incidence rate for cohort *h* corrected for historically improving 5-year survival rates, SUR(*h*,*t*), shown here for lower GI tract cancer for two birth-decade cohort intervals of birth, *h* = 1890–99, and *h* = 1900–09. Applies eq. 4**.

Age at death interval, *t*	OBS(*h,t*)	OBS*(*h,t*)	SUR(*h,t*)	INC(*h,t*)	OBS*(*h,t*)	SUR(*h,t*)	INC(*h,t*)
	*h* = 1890–9	*h* = 1890–9	*h* = 1890–9	*h* = 1890–9	*h* = 1900–9	*h* = 1900–9	*h* = 1900–9
15–19	5.49E−06	5.49E−06	0.1	6.10E−06	4.96E−06	0.15	5.84E−06
20–24	1.02E−05	1.07E−05	0.1	1.19E−05	9.64E−06	0.2	1.21E−05
25–29	1.58E−05	1.59E−05	0.15	1.87E−05	1.72E−05	0.25	2.29E−05
30–34	3.02E−05	3.00E−05	0.2	3.76E−05	3.15E−05	0.3	4.50E−05
35–39	5.12E−05	5.09E−05	0.25	6.78E−05	5.54E−05	0.33	8.27E−05
40–44	1.02E−04	1.03E−04	0.3	1.47E−04	8.88E−05	0.33	1.33E−04
45–49	1.81E−04	1.83E−04	0.33	2.74E−04	1.49E−04	0.4	2.48E−04
50–54	2.99E−04	3.12E−04	0.33	4.65E−04	2.57E−04	0.4	4.28E−04
55–59	4.74E−04	4.92E−04	0.4	8.20E−04	4.46E−04	0.44	7.96E−04
60–64	7.61E−04	7.87E−04	0.4	1.31E−03	7.48E−04	0.44	1.34E−03
65–69	1.16E−03	1.22E−03	0.44	2.18E−03	1.18E−03	0.44	2.11E−03
70–74	1.69E−03	1.80E−03	0.44	3.21E−03	1.76E−03	0.44	3.14E−03
75–79	2.30E−03	2.52E−03	0.44	4.49E−03	2.44E−03	0.44	4.36E−03
80–84	3.04E−03	3.44E−03	0.44	6.15E−03	3.27E−03	0.44	5.84E−03
85–89	3.82E−03	4.60E−03	0.33	6.86E−03	4.14E−03	0.44	7.39E−03
90–94	4.46E−03	5.91E−03	0.22	7.58E−03	4.72E−03	0.33	7.04E−03
95–99	4.24E−03	6.56E−03	0.11	7.37E−03	5.51E−03	0.22	7.06E−03
100–104	2.93E−03	4.91E−03	0	4.91E−03	3.85E−03	0.11	4.33E−03

Estimation of survival rates especially in old age continues to be a source of considerable uncertainty. Here we used the values recorded in various clinical studies for American males up to age 80–84 (14) and estimated subsequent values as a linear decline to SUR(*h,t*) = 0 at age 100–104 based on discussions with clinicians about their experiences with declining use of surgery and/or other attempts at therapy in the extremely aged. The estimates of SUR(*h,t*) used for the birth cohorts of 1890–99 and 1900–99 are listed in Table 2 so that readers may understand this process and that our estimates of SUR(*h,t*) for ages 85–104 are “best guesses.”

#### Test cohort: EAM lower GI tract cancer mortality in the U.S., 1900–2006

Deaths from cancer of the lower gastrointestinal tract (present ICD9 codes: 152, 153, 154) are predominantly from colorectal cancer (see text footnote 1). Figure 1A uses a semi-log plot to illustrate the transformations from raw mortality rates to incidence rates: from OBS(*h* = 1890–99, *t*) to OBS^∗^(*h* = 1890–99, *t*), then to INC(*h* = 1890–99, *t*). Also shown is the result of the same transform of the data of the birth cohort of the succeeding decade, INC(*h* = 1900–09, *t*), which yielded similar estimates of for all values of “*t*” (Table 2).

In Figure 1B the coincidence adjusted mortality rate, OBS^∗^(*h*,*t*) is presented as a function of age of death, *t*, on a semi-log scale for each of successive birth cohort intervals, “*h*,” from 1800–09 through 2000–06. These data demonstrate that colorectal cancer death rates in older adults (*t* > 65) rose throughout the birth cohorts of the nineteenth century reaching an approximately stable maximum in and after the birth cohort of 1880–89 modified to the extent discussed above by improvements in medical treatment represented by increasing values of SUR(*h*,*y*).

In Figure 1C these same data for colorectal cancer are plotted so that changes OBS^∗^(*h*,*y*) as a function of age of death interval, “*t*” may be seen as functions of historical year of death interval “*y.*” Here one may clearly see the historical changes in successive birth cohorts as maximum mortality rates rose for unknown reasons in cohorts born in the early nineteenth century and declined steadily for cohorts living in the twentieth century when improved surgical procedures increased survival rates.

In Figure 1D the data are plotted as OBS^∗^(*y,t*) vs. “*y*” to illustrate the historical changes within each age of death interval, “*t*” as a function of historical year of death interval, “*y*.” This form of presentation shows a significant decrease in older adult death rates ascribable in whole or part to advances in medical practice after 1950 that increasingly effect each successive birth cohort (Table 2) and a marked, previously unrecognized and unexplained decrease in pediatric death rates dating from ∼1940.

### Amendments of the Armitage–Doll “two-stage” cancer model

#### Limitation of initiation mutations to the fetal/juvenile stem cell doublings

Growth of normal fetal/juvenile stem cells is here modeled as a series of “*a*” net binomial doublings (*a* = 0, 1, 2, …, *a*_max_) defining the growth of the number of stem cells in an organ, *N*(*a*), throughout fetal and juvenile growth to maturity. This does not mean that we assume that each and every fetal/juvenile cell survives and grows exponentially by binomial fissions. We are aware that many organs, e.g., lung, breast, prostate, vascular system, grow as arborated ductal structures. But we note that their net growth may be reasonably approximated as a binomial expansion. Our mutation rates are in essence the probability that any new cell in the stem cell expansion resulting in a mature organ has newly acquired a required oncomutation (event) in the net cell doubling interval represented by “*a*.”

The number of fetal stem cells during growth *N*(*a*) is thus simply represented as *N*(*a*) = 2*^a^*. Initiation is postulated to occur in any stem cell by acquisition “*n*” required initiation mutations, *i*, *j*, …, *n*, occurring in any order at constant mutation rates *R*_i_, *R*_j_, …, *R*_n_ per doubling (14, 20). The expected number of newly initiated stem cells in each doubling period “*a*,” NEW_init_(*a*) may be expressed as:

(5) $$\begin{array}{cc} \mathrm{NE}W_{\mathrm{init}}\left(a\right)=n\left(\prod_{n}R_{i}\right)a^{\left(n-1\right)}2^{a} & 0\leq a\leq a_{\max} \end{array}$$

In the fetal/juvenile model organogenic stem cells are posited to reach maturity represented by “*a*_max_” doublings with high constant mutation rates and to undergo metamorphosis to maintenance stem cells with no net additional net cell growth and much lower mutation rates (6).

Assuming each of the ∼10^7^ adult colonic crypts to be represented at juvenile/adult metamorphosis by a single metakaryotic stem cell, the number of net doublings at maturity, *a*_max_, is about 23.25, i.e., 10^7^ ∼2^23.25^ (14, 20). The metakaryotic mutator/hypermutable stem cell lineage of human organ anlagen is here formally postulated to begin in gestational week 4–5 with creation of two metakaryotic stem cells from symmetrical amitosis of a single precursor embryonic mitotic stem cell at *a* = 0 (4). At birth, we estimate that a human colon contains ∼2^20^ colonic crypts each containing a basal metakaryotic stem cell; thus at birth, *a* ∼20, at maturity, *a* = *a*_max_ ∼ 23.25.

#### Promotion mutations during preneoplastic stem cell doublings

While the age of the developing organ may be designated as “*a*,” each initiated stem cell arises as a single cell and is here posited to double in parallel with its organogenic lineage till *a*_max_ and then continue to grow in adult tissue at a rate of doubling, μ, similar to that of the juvenile organ in which it resided (14). At maturity, initiated colonies arising early in fetal growth will be larger than those initiated at the last pre-maturation doubling that would consist of a single initiated stem cell. (30, 31) Each initiated colony is posited to grow at a constant rate, μ, until one preneoplastic stem cell experiences “*m*” required events to promote it to a neoplastic stem cell that founds a rapidly growing, potentially lethal adenocarcinoma with sequelae, including metastasis, leading to death. Here we adopt the suggestion of Armitage and Doll (1) that the time between promotion and death is about 2.5 years in adults.

#### Transforming stem cell doublings, *g*, into human age, *t*

In order to express the idea that initiated colonies arise throughout fetal/juvenile growth of organogenic stem cells and continue to grow in adult tissues we needed to define a continuous variable that expressed the age and, therefore the size, of each preneoplastic lesion throughout both fetal/juvenile and adult life.

To this end we introduce the continuous variable “*g*” that is equal to “*a*” for all values of “*a*” from *a* = 0 to *a* = *a*_max_ and then increases by one (1) for each adult preneoplastic stem cell doubling period in years represented by 1/μ where μ is the doubling rate of stem cells in an adult preneoplasia. Assuming an age of organ maturity as ∼16.5 for males and a 2.5-year interval between a promotion event in an initiated preneoplastic stem cell and death, the relationship between age in years, *t*, and age in terms of stem cell doublings since the beginning of organogenesis when *a* = *g* = 0 is simply:

(6) $$g=\mu \left(t-16.5-2.5\right)+a_{\max}=\mu \left(t-19\right)+a_{\max}$$

This introduction of the parameter “*g*” is essential to our modeling approach in that it provides a means to relate events driven by stem cell doubling intervals, “*g*,” to mortality rates recorded in age of death intervals in adult life, “*t*.”

#### Assembling the elements into a single continuous model of cancer incidence

After initiation in any fetal/juvenile doubling “*a*” growth of preneoplastic stem cells as a colony is modeled as a series of “(*g* − a)” binomial doublings [(*g* − *a*) = 0, 1, 2, …]. In each preneoplastic stem cell doubling events such as mutations may occur until any preneoplastic stem cell experiences “*m*” required promotion events (*A*, *B*, …*m*). These events are posited to occur at constant mutation rates per doubling: *R*_A_, *R*_B_, …, *R*_m_. The expected number of newly initiated stem cells in preneoplastic doubling period “(*g* − *a*)” NEW_prom_(*g* − *a*), is simply:

(7) $$\begin{array}{llll} & \mathrm{NE}W_{\mathrm{prom}}\left(g-a\right)\; & & \;\\ & \;=\;m\left(\prod_{m}R_{A}\right){\left(g-a\right)}^{\left(m-1\right)}2^{\left(g-a\right)}\;0\leq \left(g-a\right) \end{array}$$

Under these assumptions the number of organogenic doublings “*a*” at initiation and the number of preneoplastic doublings “(*g* − *a*)” after initiation sum to “*g*.” In each organogenic-doubling interval “*a*” new preneoplastic colonies are created (initiated) and these colonies grow until promotion and subsequent death remove them.

For each organogenic stem cell doubling period “*a*” we now require an expression for the expected number of colonies per individual that are promoted in any interval of (*g* − *a*) thereafter. Since the promotion of a preneoplastic stem leads to death the number of surviving preneoplastic after initiation in interval “*a*” will decline in each interval (*g* − *a*).

For the set of initiated colonies arising in organogenic interval “*a*” the expected number of promotions in the subsequent lifetime intervals, *g*, EXP(*a*  → *g*) may be expressed as:

(8) $$\begin{array}{llll} \mathrm{EXP}\left(a\to g\right) & =n\left(\prod_{n}R_{i}\right)a^{\left(n-1\right)}2^{a}d\left[1-\exp\left(-m\left(\prod_{m}R_{A}\right)\right.\right.\; & & \;\\ & \;\left.\left.{\left(g-a\right)}^{\left(m-1\right)}2^{\left(g-a\right)}\right)\right]∕d\left(g-a\right)\; \end{array}$$

While this expression may be computed for all values of “*n*” and “*m*” and is used in our computations for varying values of “*n*” and “*m*” below, we here introduce the restriction of *n* = 2 and *m* = 1 for the purpose of clearer illustration.

(9) $$\begin{array}{llll} \mathrm{EXP}\left(a\to g\right) & =2R_{i}R_{j}a2^{a}d\left[1-\exp\left(-R_{A}\;2^{\left(g-a\right)}\right)\right]∕d\left(g-a\right)\; & & \;\\ & =2\ln2R_{i}\;R_{j}R_{A}2^{g}a\;\exp\left(-R_{A}2^{\left(g-a\right)}\right) \end{array}$$

Of course, the expected number of promotions to neoplasia in an adult in any interval “*g*,” *V* (*a* → *g*), is simply the sum of expected promotions to neoplasia from the initiations of organogenic stem cells in each of the organogenic stem cell doubling intervals of *a* = 0 to *a* = *a*_max_:

(10) $$\begin{array}{llll} & V\left(a\to g\right)\; & & \;\\ & \;=\sum \left(a=0\;\mathrm{to}\;a_{\max}\right)\;2\ln2R_{i}R_{j}R_{A}\;2^{g}a\;\exp\left(-R_{A}2^{\left(g-a\right)}\right) \end{array}$$

**Figure 2:**
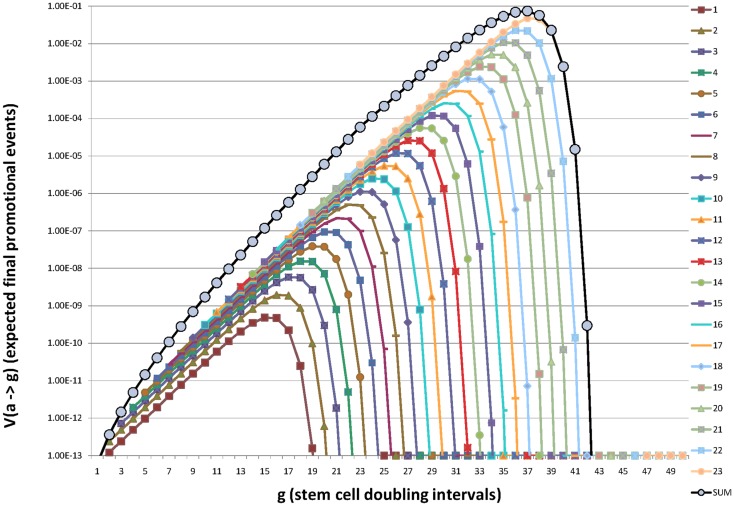
**Illustration of how initiation of organogenic stem cells in each successive stem cell doubling during fetal/juvenile growth to maturation contributes additively to the expectation of promotion (and death) throughout life**. Here the beginning of organogenesis is represented as *a* = *g* = 0, birth is at approximately, *a* = *g* = 20, and maturity is at *a* = *a*_max_ = *g* = 23.25. For a preneoplastic stem cell growth rate of μ = 0.2 the maximum value of *V* (*a*  → *g*) is reached at *g* = 37 or about or about *t* = 90 years and *g* = 40 corresponds to *t* ∼ 105 years. Note that earliest initiations presage fetal, juvenile, and young adult deaths. Initiations late in organ development account for deaths in extreme old age. Note the expected number of promotions (deaths), *V* (*a*  → *g*) increases sub-exponentially from *g* = 23 to a maximum at *g* ∼ 37 and then declines rapidly. In this example *g* = 40 represents ∼105 years of age. Values used in this illustration were *n* = 2, *m* = 1, *R*_i_ = *R*_j_ = 2.2 × 10^−5^, and *R*_A_ = 4.4 × 10^−5^.

This process is illustrated in Figure 2 in which the contribution to promotion at age “*g*” from initiation at each organogenic doubling “*a*” is shown to rise and fall with “(*g* − *a*).”

Figure 2 embodies our central argument that initiation is restricted to the metakaryotic stem cell doublings of organogenesis in the fetal/juvenile period.

The sum of these terms from initiations in all organogenic-doubling intervals “*a*” approximates well the observed lifetime incidence rate of many cancer types including colorectal cancer: it increases sub-exponentially, reaches a maximum in old age and declines appreciably in extreme old age. The earliest initiations of fetal organogenesis drive the tumor incidence rate of juveniles and young adults, the initiations of adolescent organogenesis drive the tumor incidence rate in extreme old age (31).

Under these conditions the expected number of newly promoted lesions through the end of any adult lifetime doubling interval “*g*,” CAL(*g*), is:

(11) $$\mathrm{CAL}\left(g\right)=\left(1-e^{-V\left(a\to g\right)}\right)$$

#### Additional possible modeling elements

##### Stratification of risks in the population

Stratification, or differences, within the population may encompass any environmental or heritable condition required for cancer death. Some environmental risks appear to operate through growth stimulation of preneoplastic colonies as opposed to induction of initiating or promoting oncomutations, e.g., persons exposed to sunlight as adults or adult cigarette smokers. Populations may thus be “stratified” on the basis of exposure to such preneoplastic growth-promoting agents.

Other risks may well be stratified including fetal/juvenile initiation mutation rates (6, 32), promotion mutation rates, growth of promoted neoplastic stem cells (tumor progression), and tumor invasion/metastasis. It is well to note that mortality data modeling provides information about the subpopulation at risk for a form of cancer but nothing about persons who are not at risk.

We have previously represented the fraction of the population in whom all of the potential conditions necessary for cancer death are present as “*F*” imagining that a person either is or is not at risk for all necessary oncogenic processes. The corresponding fraction in which one or more necessary condition(s) of risk is absent has been represented as (1 − *F*) (14, 20). Stratification need not, however, be an “all or none” phenomenon. Children grow to different sizes, which may create stratification with regard to the maximum number of stem cells at risk of initiation $2^{a_{\max}}$. Children also grow at different rates and so may the preneoplastic lesions in different persons. We have reported stratification with regard to mutation rates in fetal/juvenile growth for both mitochondrial and nuclear genes (9). In progress is an effort to incorporate stratification with regard to initiation and promotion oncomutation rates and the growth rates of preneoplasias. Here we continue to employ “*F*” as an approximation for more precise stratification variables. Equation 11 rewritten to account for stratification in this way creates the model:

(12) $$\mathrm{CAL}\left(g\right)=F\left(1-e^{-V\left(a\to g\right)}\right)∕\left[F+\left(1-F\right)e^{{\int \;}_{V\left(a\to g\right)\mathrm{dg}}}\right]$$

##### Competing forms of risk potentially affecting age-specific cancer rates

Epidemiological observations have also demonstrated that forms of cancer may share environmental or inherited risk factors with another, e.g., breast and ovarian cancers, in which the death rates increase synchronously with age (see text footnote 1). The term “*f*” has been previously introduced to represent the fraction of persons that die of the observed cause among the set of mortal diseases with shared risks and synchronous changes in death rates (14, 20). We considered it possible that vascular disease death rates that rise synchronously with cancer death rates might represent a major form of competing risk. However, inspection of the stable and then declining death rates from cerebrovascular and cardiovascular death rates 1900–2006 while colorectal cancer rates have increased to stable maxima (accounting for increased survival) indicates that risks for vascular disease and colorectal cancer are probably not shared (see text footnote 1). Herein, treating colorectal cancer specifically, we have assumed *pro tempore* that there are no synchronously competing forms of death with shared risks for colorectal cancer (34), i.e., *f* = 1.0. As noted, this assumption is not valid for other forms of cancer such as breast, uterine, and ovarian cancers, which appear to share risk factors.

### A computer program to estimate parameters of carcinogenesis in the two-stage model adjusted for fetal/juvenile initiation: CancerFit v5.0

#### Using CancerFit v5.0

In order to permit cancer clinicians and biologists to explore quantitative hypotheses linking a wide range of biological and population parameters to observed lifetime incidence rates the computer program CancerFit v5.0 was developed and is available without cost from the corresponding author or by download from http://mortalityanalysis.mit.edu. Any computer carrying MatLab©v.7.14 can run this program. Data for any form of cancer downloaded from http://mortalityanalysis.mit.edu as OBS^∗^(*h,t*) and be directly imported into the program for the desired country (U.S. or Japan), gender, ethnic group, and birth year interval. Values of the parameters (Π*R*_i_)^1/^*^n^*, (Π*R*_A_)^1/^*^m^*, μ, and *F* may be either fixed or permitted to range with a chosen number of iterations for computation within each parameter range chosen by the researcher. On a typical 2009 computer such as a MacIntosh G5^®^ 10^9^ iterations of parameter combinations can be run in about 4 h. In typical use the combinations of variable iterations may range from 20^4^ to 20^5^ (1.6 × 10^5^ to 3.2 × 10^6^) requiring a few minutes for computation. The results of a single computation run are presented as the set of 100 best fits of the stipulated model to the values of INC(*h,t*) as defined below.

The researcher may then inspect the ranges of values of all five parameters that together result in calculated age-specific incidence estimate CAL(*h,t*) in good agreement with observation, INC(*h,t*) for the stipulated values of “*n*,” “*m*,” and “*a*_max_.” The tabulated results also provide the estimated parametric values for the fraction of persons initiated, *F*_init_, and the area computed under the function CAL(*h,t*), AREA(*h*), from *g* (or *t*) = 0 to ∼150 years, which roughly estimates the expected (potential) number of lethal events from the observed disease per person over the lifetime of the cohort. Graphic representations are provided comparing CAL(*h,t*) to INC(*h,t*) for any desired fit in terms of linear or log values of INC(*h,t*) vs. linear or log values of age of death, “*t*.” It is hoped that this program and the organized set of U.S. and Japanese mortality data for a large set of cancer sites (see text footnote 1) will enable cancer researchers and students to explore quantitative hypotheses about cancer etiology from both a historical and age-specific perspective for the genders and ethnic groups represented.

#### Ranges of parameter values employed in calculating values of CAL(*h,t*) for colorectal cancer

The central premise of the fetal/juvenile mutator/hypermutable stem cell hypothesis is that initiation mutations occur before adulthood when the maximum number of juvenile stem cells, $2^{a_{\max}}$ is reached, i.e., 0 ≤ *a* ≤ *a*_max_ (6, 35, 36). In CancerFit v5.0 the value of *a*_max_ must be specified for each organ studied, here we used 23.25 as an estimate for the colon/rectum (14). The values of *F* must lie between 0.0 and 1.0; for computation of CAL(*h,t*) 20 values, 0.05, 0.1, …, 0.95, 1.0, were used for each. Combinations of *n* = 1–5 and *m* = 1–5 were independently explored. Values of μ, the preneoplastic annual net growth rate, ranged with 20 linear iterations from 0.1 to 0.3 bracketing estimates from the annual growth rates of juvenile body mass, ∼0.16 (14, 20). Values of (Π*R*_i_)^1/^*^n^* representing the geometrical average of required oncomutation rates in initiation and of (Π*R*_A_)^1/^*^m^* representing the geometrical time-averaged required onco-event rates in promotion ranged from 10^−9^ to 10^0^ with 100 geometric iterations. After these geometrical means were approximated, the values were bracketed more closely to asymptotically obtain the best fits for each combination of *n* and *m* tested.

#### Goodness of fit

The values of INC(*h,t*) vary more than a thousand-fold among the 18 age of death intervals recorded from maturity to old age (Figure 1). Statistical comparison of CAL(*h,t*) to INC(*h,t*) required a term that gave equal weight to all 18 intervals. Such a term is (log_10_ CAL(*h,t*) − log_10_ INC(*h,t*)(that is zero when the terms are identical, i.e., their ratio is equal to 1.0. The square root of the average of the square of these 18 terms is here employed as a goodness of fit parameter, GOF(*h,t*).

(13) $$\mathrm{GOF}\left(h,t\right)={\left\{\sum {\left[\log_{10}\;\mathrm{CAL}\left(h,t\right)-\log_{10}\;\mathrm{INC}\left(h,t\right)\right]}^{2}∕18\right\}}^{1∕2}$$

GOF(*h,t*) is thus akin to a standard deviation of (log_10_ CAL(*h,t*) − log_10_ INC(*h,t*)(averaged over all adult life. If GOF(*h,t*) = 0.1 then the 95% confidence limits of the average ratio of CAL(*h,t*)/INC(*h,t*) would be 10^−0.2^ and 10^0.2^ or 0.63 and 1.58. If GOF(*h,t*) = 0.03 the 95% limits would be 0.93 and 1.07. A GOF(*h,t*) of greater than 0.1 suggests a poor fit of the model with stipulated parameters “*n*” and “*m*” or marked errors in the values of INC(*h,t*) arising from either sampling error or bias. Errors arising from small sample sizes do not contribute significantly to GOF(*h,t*) when large U.S. European-American cohorts are studied. In the death intervals containing the fewest recorded deaths the number of lower GI tract European-American male cancer deaths recorded for the birth cohort of 1890–99 were 246 for *t* = 15–19 (1908–1917) and 1999 for *t* = 100–104 (1993–2002) respectively (see text footnote 1). Based on the assumption of a Poisson distribution of deaths per living person within each age of death interval, *t*, the GOF(*h,t*) for two random samples of the same population would yield an expected GOF(*h,t*) of about 0.03.

Given the various biases and sampling errors expected for measurements of OBS(*h,t*), TOT(*h,t*), and especially SUR(*h,t*) defining the derived function INC(*h,t*) this would be as good a fit as might be expected for any model. When the values of INC(*h* = 1890–99, *t*) were compared to INC(*h* = 1880–89, *t*) and INC(*h* = 1900–09, *t*) over all 18 age of death intervals GOF(*t*) was ∼0.043 a number not much greater than the value ∼0.03 expected by chance alone (Table 3).

**Table 3:** **Matrix of goodness of fit calculated as GOF(*h,t*) for *n* = 1, 2, 3, 4, 5 and *m* = 1, 2, 3, 4, 5 under the parsimonious conditions of homogeneous risk (*F* = 1) and no synchronous mortal diseases sharing risk factors with colorectal cancer (*f* = 1)**.

	*n*					
		1	2	3	4	5
	1	0.066	0.085	0.098	0.128	0.094
	2	0.072	0.079	0.084	0.052	0.074
***m***	3	0.064	0.089	0.078	0.055	0.089
	4	0.098	0.109	0.098	0.071	0.103
	5	0.088	0.107	0.114	0.087	0.110

Values of the geometric means of initiating mutation rates (∏ *R*_i_)^1/^*^n^* and promotion mutation rates (∏ *R*_A_)^1/^*^m^* were permitted to range from 10^−9^ to 10^ 0^ and the range of μ was set at 0.1–0.3. The results illustrate the fact that values of *n* and/or *m* cannot be derived by simply fitting the non-linear function, CAL(*h,t*) represented by the algebraic model to the data, INC(*h,t*): multiple combinations of *n* and *m* result in values of GOF(*h,t*) indicating reasonable correspondence of the model to the data.

## Results

### Application of the model to age-specific colorectal cancer incidence in a specific cohort

First, the best fits of CAL(*h* = 1890–99, 15 < *t* < 104) were calculated for the 25 combinations of *n* = 1–5 and *m* = 1–5 under the parsimonious conditions of homogeneous risk and no synchronous mortal diseases sharing risk factors with colorectal cancer (*F* = 1, *f* = 1). Values of (Π*R*_i_)^1/^*^n^* and (Π*R*_A_)^1/^*^m^* were permitted to range from 10^−9^ to 10^0^ and the range of μ was set at 0.1–0.3. The complete matrix of results is provided in Table 3. For *n* = 2 (an appropriate biological value only if the loss of both copies of the *APC* gene were necessary and sufficient for initiation in most colorectal cancers) and *m* = 1 as default assumptions the GOF(*h,t*) was 0.085.

Second, we considered the possibility that stratification of risks within the population such as varying oncomutation rates might account for the observed discordance (*F*, 1, *f* = 1). To do this we compared CAL(*h,t*) to INC(*h,t*) under the additional assumption of inhomogeneous risk(s), i.e., the parameter “*F*” representing a hypothetical fraction of the population at risk was allowed to range from 0 to 1. The value of 0.043 was the minimum GOF(*h,t*) observed for *n* = 2, *m* = 1 and the concordance in the age range 80–104 was appreciably improved. The complete matrix of these results is provided as Table 4.

**Table 4:** **Complete matrix of GOF(*h,t*) for *m* = 1, 2, 3, 4, 5 and *n* = 1,2,3,4,5 with the assumption of inhomogeneous risk, i.e., the parameter “*F*” representing a hypothetical fraction of the population at risk was allowed to range from 0 to 1 (*F* < 1) and no synchronous mortal diseases sharing risk factors with colorectal cancer (*f* = 1)**.

	*n*					
		1	2	3	4	5
	1	0.038	0.043	0.039	0.041	0.046
	2	0.036	0.048	0.042	0.046	0.054
***m***	3	0.043	0.042	0.046	0.042	0.043
	4	0.047	0.047	0.035	0.040	0.044
	5	0.051	0.050	0.064	0.047	0.045

Values of (∏ *R*_i_)^1/^*^n^* and (∏ *R*_A_)^1/^*^m^* were permitted to range from 10^−9^ to 10^0^ and the range of μ was set at 0.1–0.3. Comparison with the values of GOF(*h,t*) of Table 3 illustrates that the assumption of population inhomogeneity results in better agreement between model and incidence data. Such inhomogeneity has been reported as an appreciable range of fetal juvenile mutation rates for the human lung bronchial epithelium (6).

Thirdly, we considered the possibility of both population inhomogeneity and a competing synchronous mortal disease having genetic and/or environmental risks shared with colorectal cancer (*F* < 1, *f* < 1). This assumption did not, however, further reduce the values of GOF(*h,t*) consistent with the assumption that other appreciable forms of mortality do not share risk factors with lower G.I. tract cancers (34).

**Figure 3:**
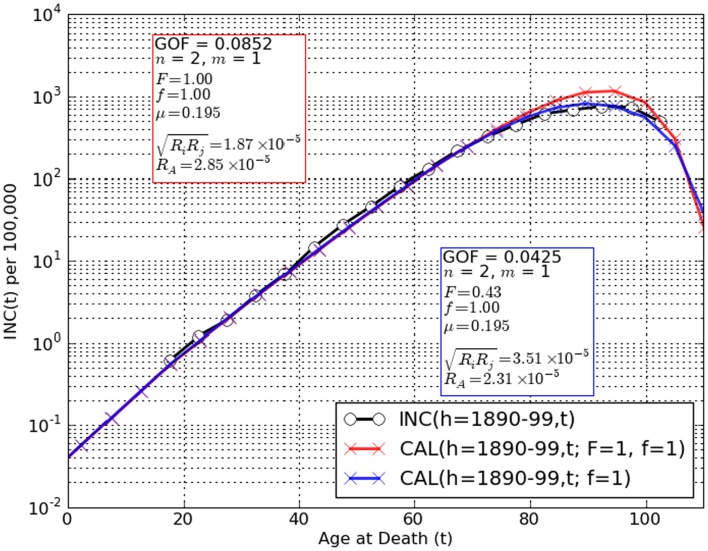
**Comparisons of observation INC(*h,t*) (black circles) to CAL(*h,t*) (red, blue crosses) for the two-stage cancer model amended for limiting tumor initiation mutations to the fetal/juvenile stem cells and *n* = 2, *m* = 1**. A remarkably good fit is obtained for the assumptions that the population is homogeneous for risk factors such as oncomutation rates and/or preneoplastic growth rates (*F* = 1) and there are no competing synchronous forms of death (*f* = 1) [red crosses, GOF(*h,t*) = 0.085]. However, a significantly lower value, GOF(*h,t*) = 0.043 is observed when population risk stratification is assumed (*F* < 1). Allowing for the possibility of an unknown competing synchronous form of death (*f* < 1) did not decrease the values of GOF(*h,t*) further. These findings are spurring further amendment of the two-stage model to use stratified variables for initiation and promotion mutation rates, preneoplastic growth rate and number of stem cells at maturity.

Figure 3 graphically depicts the degree of concordance of the trial conditions: *F* = 1 (population homogeneity with regard to risk) and *F* < 1 (population inhomogeneity) with adult lifetime incidence data for lower G.I. tract cancer in European-American males born 1890–99 INC(*h,t*). It should be noted that for the assumption *F* = 1 discordance between INC(*h,t*) and CAL(*h,t*) was greatest at *t* > 75 years where underestimation of colorectal cancer as a cause of death by as much as 30% has been suspected in extreme old age (14, 20, 21, 37, 38). Note that our tactic of converting the raw mortality rate estimate OBS(*h,t*) to the coincidental death corrected estimate OBS^∗^(*h,t*) would not account for under-diagnosis post mortem. However, when CAL(*h,t*) accounts both for fetal/juvenile mutation limitation (6) and the *possibility* of risk stratification in the population (*F* < 1) fits are considerably improved (Figure 3).

### Use of observations in humans to estimate number of events required for initiation (*n*) and promotion (*m*) in colorectal cancer

Comparison of the predictions of the amended Armitage–Doll two-stage model represented as CAL(*h,t*) to the observed lifetime adult age-specific incidence of colorectal cancer in European-American males born 1890–99 or 1900–09, INC(*h,t*) achieved a higher degree of concordance over the entire adult lifespan than has previously been reported for any previous model of human cancer. This result is consistent with the hypothesis that tumor initiation mutations are limited to the stem cells of the fetal/juvenile period and that there is a significant degree of heterogeneity of risk factors in the population (*F* < 1). We have reported considerable variation in the population with regard to fetal/juvenile mutation rates in the human lung (6). However, as noted by Peto, no determination of the values of the number of initiating (*n*) or promoting (*m*) events is possible by this approach alone as evidenced by the good fits for all 25 combinations of *n* and *m* tried (Table 4) (33).

However, we could now apply several observations delimiting some of the key parameters and discover what, if anything, may be suggested here using the stipulations that *F* < 1, *f* = 1.

First, we applied our observation that juvenile colonic crypts each contain a single metakaryotic stem cell (4) and that the number of crypts in an adult male colon is about 10^7^ or 2^23.25^ (14). This value we associated with the maximum number of juvenile colonic stem cells before maturation to adult colonic maintenance stem cells with a form of eukaryotic behavior, i.e., we estimated that *a*_max_ = 23.25.

Second, we again noted that colonic fetal/juvenile mutation rate estimates of ∼2–5 × 10^−5^ gene-inactivation mutations per juvenile stem cell doubling have been derived from observation of adenomatous polyps or mutations in the colon for the *APC* and *OAT* genes (11, 12). Independent of the value of *m* stipulated, the only value of *n* that resulted in such an initiation mutation rate range is 2. For *n* = 2 the estimated rate was ∼3.5 × 10^−5^; for *n* = 1, ∼5.7 × 10^−8^; for *n* = 3, ∼3.6 × 10^−4^.

The provision of the fetal/juvenile stem cell mutation rate thus permitted the conclusion that *n* = 2 events. These could comprise loss of both maternal and paternal *APC* alleles in the numerically dominant colorectal cancer pathway (24).

Third, we previously noted the annual juvenile mass doubling rate of U.S. males is about 0.158, significantly lower than estimates of preneoplastic growth rates of 0.18–2.0 generated when it is assumed that *n* = 2 and *m* = 1 (14).

Fourth, we estimated loss of heterozygosity fractions for many different polymorphic markers in colon tumors are about 0.125 for a single alleles (14). With preneoplastic doubling rates estimated to be between 0.158 and 0.2 the number of years between male maturity and the age at which mortality rate reaches a maximum represents about 15 doublings. We here made a rough estimate from these values: 0.125/15 = 8 × 10^−3^ gene deletion events per doubling. This represented a lower bound on gene-inactivation or loss rates in preneoplastic stem cells. If loss of either allele for an autosomal gene were a promotional event the rate would be twice this estimate or >1.6 × 10^−2^.

As the values of *m* from 1 to 5 (holding *n* = 2) increased, the geometric mean of the promotion event (mutation) rates increased. For *m* = 1 the estimate was 2.2 × 10^−5^ similar to the estimated rate of promotion events; for *m* = 2, ∼1.9 × 10^−3^; for *m* = 3, 7 × 10^−3^; for *m* = 4, 1.4 × 10^−2^; for *m* = 5, ∼2.1 × 10^−2^.

As the values of *m* increased the estimated growth rate of preneoplastic stem cells decreased. A decrease in estimated preneoplastic doubling rates with increasing values of *m* was expected. For values of *m* > 1, the generating function for promotion would rise as (*g* − *a*)^(^*^m^*^ − 1)^ as originally imagined by the earliest cancer modelers (15–17). This supralinear term would force a decrease in the estimate of the exponential growth rate of preneoplastic stem cells, μ, in order to fit the data of INC(*h,t*).

We noted that for *m* = 3, μ = 0.167, and the geometrical mean of the three promotion event rates, (Π*R*_A_)^1/^*^m^* was ∼7 × 10^−3^. For *m* = 4, μ = 0.162, and (ΠR_A_)^1/^*^m^* = 1.4 × 10^−2^. For *m* = 5, μ = 0.156, and (Π*R*_A_)^1/^*^m^* = 2.1 × 10^−2^. Given the several uncertainties in the estimate of preneoplastic gene losses and the range observed among losses, it seemed that values of *m* = 4 or 5 would best fit the data. These observations further suggest that the growth rate of preneoplastic stem cells in males is indistinguishable from the juvenile growth rate of males, ∼0.158 (14).

## Discussion

### Fetal/juvenile mutation

Early cancer models posited that a constant number of adult cells were at continuous risk of acquisition of a required set of oncomutations that resulted in lethal tumors (15–17). Platt’s questions about selective growth advantage of early oncomutants (18) stimulated the creation of the two-stage model of Armitage and Doll in which initiation was restricted to a constant number of adult cells (1). An alternate theory, fetal/juvenile or, more narrowly, gestational initiation was based on Luria’s demonstration that in a bacterial culture growing from a small number of cells early mutations give rise to large mutant colonies and late mutations to small ones (10). This concept was clearly applicable to the stem cells of a growing organ. By maturity a stem cell initiated early in development would create a large preneoplastic colony, an initiation in the last juvenile doubling would create a single initiated stem cell. Early cancers would arise from early developmental initiation, cancers of extreme old age would arise from the last juvenile initiation events (30, 31, 39, 40). In the algebraic model provided here each successive net doublings of stem cells of the fetal/juvenile period provides initiated colonies. Deaths from colonies initiated in any specific doubling interval would be distributed over subsequent years of life until all persons with initiated colonies have died (Figure 2).

The Armitage–Doll two-stage model, CAL(*h,t*), of age-specific cancer rates amended to incorporate a (a) defined maximum number of pre-maturation colonic epithelial stem cells and (b) limitation of tumor initiating mutations to the fetal/juvenile period accurately predicts the observed incidence function INC(*h,t*) for colorectal cancer in the cohort examined.

Biological data supporting limitation of mutations to the fetal/juvenile period were scant but suggestive. Brash and Ponten reported that increases particular point mutant *TP53* colonies in human skin were restricted to the juvenile years and that subsequent solar exposure in adults increased mutant colony size but not number (35, 36). More recently, the distributions of five nuclear and 17 mitochondrial point mutations in adult human lung epithelium were found to match a simple Luria–Delbruck expansion of mutant stem cells for ten stem cell doublings prior to maturity. Both the nuclear and mitochondrial mutant fractions assayed in human lung epithelium remained constant over the age range of patients, 35–76 years (6).

Consistent with the fetal/juvenile limitation of tumor initiating mutations are the findings that (a) sunlight does not induce new nuclear mutations in adult skin (35, 36) and (b) cigarette smoke does not induce new nuclear or mitochondrial mutations in adult human lung epithelium (6, 9). There appear to be no data indicating that mutant fractions increase in adult solid organs (41) although increases with age in circulating leukocytes have been reported, see (6) for review. This requires rethinking about human carcinogenesis in which it is generally believed (and taught) that environmental mutagens continuously initiate tumors by acting on adult stem cells despite the absence of any supportive data.

### Mutator/hypermutable metakaryotic stem cells of organogenesis and carcinogenesis

The observed high fetal/juvenile mutation rates have been associated with amitotic, non-eukaryotic cells that arise in the fourth- to fifth-week of gestation that appear to serve as the stem cells of human organogenesis and carcinogenesis (2, 3, 6, 32). These large “metakaryotic” mononuclear cells or syncytia with bell shaped nuclei are found in all human fetal/juvenile organs, preneoplastic lesions, and neoplasias. They increase by symmetric amitotic fission and have been observed to produce all parenchymal, subsequently mitotic, cell forms by asymmetrical mitotic fissions in tissues derived from all three primordial germ layers (4). Metakaryotes also display bizarre modes of chromatin organization (5) and of DNA segregation that occurs prior to, or concomitant with, DNA replication in sister cells (4). Curiously, the replication process appears to involve DNA copying by the error-prone DNA polymerase β insofar as about half of cancer-initiating *APC* point mutation hotspots sampled are attributable to errors of DNA polymerase β copying undamaged DNA *in vitro* (42).

### Number (*n*) and rates of required initiating mutations

Within the algebraic expression of CAL(*h,t*) the term (Π*R*_i_)^1/^*^n^* represents the geometrical mean of “*n*” initiating mutations. Estimates of this parameter derived from best fits of the model given the assumption of population stratification for *n* = 2 and *m* = 1–5 were 3.5, 2.3, 2.8, 2.8, and 4.3 × 10^−5^ respectively (Figure 3) agreeing well with estimates from clinical enumeration of *APC*^−/−^ mutant polyps and *OAT* gene-inactivating mutation rates of ∼2–4 × 10^−5^ per stem cell doubling during colon organogenesis (11, 12). For *n* = 1, *m* = 1, *F* < 1, the rate estimate of (ΠR_i_)^1/^*^n^* is about 5.3 × 10^−8^, for *n* = 3, *m* = 1, *F* < 1 about 2.8 × 10^−4^. Values of the geometrical mean of initiation mutations assuming values of *n* other than 2 are thus clearly discordant with *APC* and *OAT* colonic mutation rate estimates. These facts, derived from clinical genetic observations in both inherited and sporadic forms of colorectal cancers are wholly consistent with the conclusion that *n* = 2 and inconsistent with values of *n* ≠ 2.

### Number (*m*) and rates of required promotion mutations

It is not mathematically possible to estimate “*m*” (Tables 3, 4) or the related geometrical mean of promotion mutation rates, (Π*R*_A_)^1/^*^m^* by simply fitting CAL(*h,t*) to INC(*h,t*).

For *n* = 2 and *m* = 1, and *F* ≤ 1, we note that the estimated values of a hypothetical promotion mutation, (Π*R*_A_)^1/^*^m^*, range from 1 to 5 × 10^−5^, approximating the estimated geometric mean rate of 2–5 × 10^−5^ calculated for gene initiating mutations in *APC* or *OAT* genes in the fetal/juvenile stem cells (11, 12).

For *n* = 2 and *m* = 4 or 5 and *F* ≤ 1 the values of (ΠR_A_)^1/^*^m^* bracket the estimated mean rate of losses of heterozygosity (LOH) of polymorphic markers in human adenocarcinomas of ∼8 × 10^−3^. This calculation depended on the assumption that LOH in colonic adenocarcinomas arose from events in preneoplastic stem cells and not for events in post promotion neoplastic stem cell divisions. This conundrum points to the need for measurements of point and larger chromosomal mutations in human colonic adenomas.

While many kinds of genetic or epigenetic events might be required in preneoplastic metakaryotic stem cells, the data suggest that oncogene activation mutations, limited to a small set of amino acid substitutions in a proto-oncogene, is an unlikely candidate. Such events are expected to occur at rates some 100 time lower than gene-inactivation rates via point mutations (43) Gene deletion pathways as represented by loss of heterozygosity of polymorphic markers could not result in oncogene activation. To date no example of a required proto-oncogene activation has been found for any major form of human cancer (6).

However, the possibility of a large number of independent promotion pathways each with lower event rates than calculated here cannot be excluded (problem of potential parallel promotion pathways). In a formal sense we cannot even exclude *m* = 0 because no genetic or other rare required event has been identified for promotion for any human tumor.

### Estimation of preneoplastic growth rate, μ

Using the best-fit stipulated values for *n* = 2, *F* < 1 and any value of *m* = 1–5, the preneoplastic growth rate “μ” was estimated to range from ∼0.192 to 0.156. This may be compared to the juvenile growth rate of mass in males, 0.158, and females, 0.163 (14) but somewhat lower than Armitage and Doll’s original estimate that death rates doubled about every 5 years, i.e., a doubling rate of 0.2 (1). For the conditions *n* = 2, *m* = 4 or 5 our estimates of μ in preneoplastic colonic adenomas brackets the estimate of male juvenile growth rates. It would seem prudent to consider the possibility that preneoplastic stem cell growth rates might even more closely approximate the juvenile growth rates than previously suggested (14).

### Environmental cancer risks during the fetal/juvenile period

Agreement between the origins of adult somatic mutations in mutator/hypermutable fetal/juvenile human stem cells and age-specific cancer rates offers an explanation of epidemiological associations between fetal and early childhood exposure to known mutagenic stimuli, particularly sunlight (35).

Generational changes of age and organ-specific cancer rates in immigrant populations toward those of the new country of residence may also be thought of in terms of fetal/juvenile initiation mutations. Adult immigrants would have experienced tumor initiation in the country of origin while children conceived and/or raised in the new country would experience mutagenic stimuli differing from those of their parents’ land. In both older and younger immigrants the environment of the new country would define promotional stresses that might act in adults by inducing promotional oncomutations and/or by selecting through growth stimulation conditionally initiated stem cells acquired in the fetal/juvenile period (44).

Similarly, the marked decrease in death rates from lower G.I. tract (Figures 1C,D) and other incurable cancers in children and young adults post 1940 may reasonably be attributed to a decline in fetal/juvenile initiation mutation rates that began circa 1940 and continued through 2006 (see text footnote 1). We note in passing that these changes began very soon after vitamin supplementation was begun in the U.S., but the constant exponential decreases persisting for more than 60 years may suggest other hypotheses.

Save for sunlight exposure of juveniles, however, there is no direct evidence that environmental mutagens cause oncomutations in humans (41). “Spontaneous” mutation caused by simple DNA polymerase misincorporation errors or errors following DNA damage by endogenous processes could account for all fetal/juvenile mutations save for juvenile mutation by sunlight. Environmental mutagens may have been or still may be indirectly responsible for some of these oncomutations. For these reasons we cannot distinguish between the hypotheses that metakaryotic stem cells of organogenesis are constitutively mutator (high spontaneous mutation rate), hypermutable (markedly susceptible to endogenous or exogenous mutagenic agents), or both.

### The assumption of population homogeneity

When it was posited that the population is in some way inhomogeneous with regard to risk, *F* < 1, the fit of the model, CAL(*h,t*) to the recorded INC(*h,t*) was significantly improved (Figure 3). One form of risk stratification is represented by the ∼tenfold range in colonic adenomatous polyp numbers observed among individuals of FAP families (11) that suggest a range of fetal/juvenile *APC* mutation rates. A similar variation of numbers of mutant crypts displaying loss of the second allele of (*OAT)* has been observed in adult colon (12). The remaining discrepancies between the two-stage model adjusted for fetal/juvenile initiation and the colorectal cancer incidence data may thus lie principally in the assumption of a homogeneous oncomutation rate that assumes a Poisson distribution of initiating events among persons in each organogenic stem cell doubling period. This oversimplification is compensated only in part by positing a value of *F* < 1 (Table 4, Figure 3).

### The assumption of synchronous competing forms of death

Assuming a competing synchronous form of death with shared risk(s) with colorectal cancer does not further increase goodness of fit under best-fit conditions (Figure 3; Table 3) in accord with epidemiological studies of familial colorectal cancer (34). This possibility must however be considered for cancers such as breast and ovarian cancers in which synchronous age-specific death rates display shared familial risk.

### The assumption of a single pathway of initiation and promotion

We and others modeling carcinogenesis have so far treated a special limited case in which there is a single or predominant pathway to a lethal cancer. Thus, for instance in the case of colorectal cancer we assume a single pathway of initiation involving mutations *i* and *j* that appear to be independent losses of the maternal and paternal alleles of the *APC* gene conjoined to a single pathway of promotion mutations A, B, C, D,…. However, it is already known that a minor pathway to colon cancer exists involving point mutations in the beta-catenin gene (45) so our calculations for initiation rates are in fact a weighted average of the APC, beta-catenin, and other unknown minor pathways.

A general case would encompass multiple initiation pathways linked to one or more promotional pathways are responsible for cancers in organs such as the lungs, prostate, breast, and pancreas for which single tumor suppressor genes have not been found.

## Summary

These quantitative analyses/ruminations point to an amended qualitative model of human carcinogenesis at least for colorectal carcinogenesis. It seems that as metakaryotic stem cells grow to form the colon they experience a very high mutation rate and that in each organogenic stem cell doubling newly initiated stem cells are created. The newly initiated cells of each stem cell doubling grow at the same rate as their sister uninitiated stem cells during the fetal/juvenile period but after maturity grow at a constant rate similar to the juvenile growth rate of about 0.16 per year. Any initiated stem cell may experience additional promotion mutations sufficient to create a first neoplastic stem cell that gives rise to a rapidly growing tumor (adenocarcinoma) that may kill *in situ* or through metastases.

If for any organogenic stem cell all necessary initiation and promotion mutations occur before maturity a pediatric tumor would be expected. But after maturity only stem cell lineages initiated before maturity are expected to grow and create the exponential increase in cancer deaths with adult age. Eventually, the subpopulation that has experienced colorectal stem cell initiation is depleted in the surviving population in extreme old age and death rates from this form of cancer in the surviving population decline.

When matched to fetal/juvenile *APC* mutation rates derived from observation of polyp number in FAP patients the number of initiation mutations, *n*, is consistent with two and only two initiation events. A value of *m* = 4 or 5 is suggested if it is assumed that gene-inactivating events occurring at rates estimate from LOH fractions in adenocarcinomas are required for promotion. Population risk stratification for initiation mutation rates is indicated by a wide distribution of mutant numbers in adult colons and by the fact accounting in part for said stratification within the amended two-stage model improves the concordance with age-specific colorectal cancer rates. These findings point to the importance of mutagenesis in fetal and juvenile stem cells as primary determinants of adult age-specific cancer rates and suggest that they account for inter-generational shifts in cancer risks among organs in immigrant populations and the observed exponential decline of recent decades in pediatric cancer mortality in solid organs such as the colon (Figure 1D).

## Conflict of Interest Statement

Our work has been supported in part by a best-effort research contract from United Therapeutics Corp. of Silver Springs, MD, USA to study metakaryotic cells in carcinogenesis. This support is listed in the acknowledgments. No payments to any co-author or expectation of patent royalties attends upon this theoretical exploration of human colorectal cancer.

## Footnotes

^1^http://mortalityanalysis.mit.edu

## Abbreviations

APC, adenomatous polyposis coli gene; FAPC, familial adenomatous polyposis coli; *OAT*, sialylmucin acid *O*-acetyl transferase gene; *t*, age interval of death; *h*, cohort (birth years, gender, ethnic group); *y*, calendar year of death; MOR(*h,t*), number of deaths; POP(*h,t*), number in population; OBS (*h,t*), raw death rate, MOR(*h,t*)/POP(*h,t*); OBS^∗^(*h,t*), raw death rate corrected for coincident deaths, corrected death rate; INC (*h,t*), corrected death rate adjusted for survival of disease to approximate incidence; CAL(*h,t*), values of incidence predicted by amended model; GOF(*h,t*), goodness of fit of CAL(*h,t*) to INC(*h,t*).
